# The SisaMob Information System: Implementation of Digital Data Collection as a Tool for Surveillance and Vector Control in the State of São Paulo

**DOI:** 10.3390/insects14040380

**Published:** 2023-04-13

**Authors:** Gerson Laurindo Barbosa, Antônio Henrique Alves Gomes, Vera Lucia Fonseca de Camargo-Neves

**Affiliations:** Epidemiology Technical Group, Department of Diseases Carried by Vectors and Intermediate Hosts, Pasteur Institute, Health Secretariat of the State of São Paulo, São Paulo 15120-000, Brazil; goreau@uol.com.br

**Keywords:** information system, application, *Aedes aegypti*, public health, epidemiological surveillance

## Abstract

**Simple Summary:**

The importance of information nowadays is a fact widely discussed and accepted and should compose the available arsenal for decision-making and the definition of strategies for disease control. The advance in the construction of the SisaWeb information system—SisaMob—represented an enhancement in the speed of obtaining and in the quality of the collected data. This allowed the universalization of the information since the data are available in report mode for public consultation or through dynamic reports in which the user selects the variables. This integration allows a better direction of vector control at the local level.

**Abstract:**

Information systems are essential instruments in managing resources, in the evaluation of the epidemiological situation, and for decision-making at all hierarchical levels. Technological advances have allowed the development of systems that meet these premises. Therefore, it is recommended to consider the optimization of data entry and its immediate georeferencing in order to obtain information in real time. To meet this objective, we describe the application introduction process for the implementation of the digital collection of primary data and its integration with the database through synchronization with the SisaWeb platform (Information System for surveillance and control of *Aedes aegypti*), developed to meet the needs of the Arbovirus Surveillance and Control Program in the state of São Paulo, Brazil. For this purpose, the application—SisaMob—was conceived in the Android Studio development environment, Google^®^, following the same guidelines as the traditional collection method. Tablets equipped with the Android^®^ operating system were used. To evaluate the implementation of the application, a semi-structured test was applied. The results highlighted that 774.9% (27) of the interviewees evaluated its use positively and, replacing the standard bulletin, 61.1% (22) of the users considered it regular to excellent. The automatic collection of geographic coordinates represented the greatest innovation in the use of the portable device, with reductions in errors and in the time taken to complete the report in the field. The integration to SisaWeb allowed obtaining information in real-time, being easily presented in tabular and graphic modes and spatially arranged through maps, making it possible to monitor the work at a distance, and allowing preliminary analyses during the data collection process. For the future, we must improve the mechanisms for assessing the effectiveness of information, increase the potential of the tool to produce more accurate analyses, which can direct actions more efficiently.

## 1. Introduction

Along with the creation of the Unified Health System in Brazil, in 1988 [1], a set of actions and services were defined to be provided by the public institutions in the three governmental realms: federal, state, and county. This way, medium- and low-complexity actions would be executed by counties, including the vectorial surveillance and control of vector-borne diseases. The state realm became responsible for structuring and managing the information systems.

Therefore, considering that the development of a good information system should attend to the needs of management and the evaluation of the epidemiological situation in all hierarchic levels, this system should be simple and decentralized. Considering the proposed objectives to achieve the activities of surveillance and control, the system should allow obtaining data in a schematic and orderly way, bringing the possibility of, among other advantages, providing support for the decision-making process [2,3].

One of the prerequisites for an information system (IS) is the software definition, which means, the application executed through a computer that brings, as a result, storage, information sharing, and report printing, as well as easy sharing.

It is also important to account the database standardization and agility in information obtained in order to integrate it into other databases. In addition, epidemiologic indexes could be generated opportunely so that local and regional managers could define immediate actions to control complaints under their responsibility.

With regard to vector-borne diseases, the occupation of space has a great influence on the levels of occurrence, especially in an urban environment; in this way, the IS must integrate the geographic positioning data related to the surveillance and vector control activities so that the epidemiological analyses pass to be three-dimensional, incorporating the geographical space and its occupation characteristics, in particular the pattern of urbanization and sanitation.

The first software developed in the State of São Paulo (SPS) was in 1993 by the Endemic Diseases Control Superintendence, autarchy of the Health Secretariat of the State of São Paulo, the governmental agency responsible for managing the arboviruses control actions in SPS until April 2022, when it was made extinct [4,5]. At that time, with the first desktop version, named SISAED, sending data from executing units to centralizing level was made with “floppy disk” storage drives. It was an improvement compared to data consolidation methods used priorly, which were made using paper forms. It is important to highlight that this software version, and the following, were developed by a multi-professional technical team from that institution, and this fact brought as an advantage the technical and operational knowledge of the activities that should be developed inside the Urban Arboviruses Surveillance and Vector Control Program (UASVCP). Based on technical knowledge in the software development area, in addition to the proximity of the team with the final user, on different hierarchic levels, it was possible to create solutions considering user needs. This made the initial testing much easier, as well as the maintenance and implementation of functional improvements, such as easing the database handling.

The development and incorporation of a web platform, called SisaWeb, became possible in 2011. This system allowed an improvement in the elaboration of tabular data, graphics, and information visualization, arranged spatially by maps. These functions facilitated data analysis and interpretation, supporting the monitoring of the UASVCP.

Furthermore, that version allowed a great amount of data to be stored and processed simultaneously by making the information available more quickly, which are important attributes of IS [2].

However, the requirement to use paper forms to collect primary data in the field and the need for trained human resources to type data on the platform did not fully achieve the principles of an information system, which are agility and opportunity.

In this article, we present the APP SisaMob, a solution for optimizing data entry on the web platform, and the possibilities of obtaining information, in real time, through the SisaWeb system.

## 2. Methodology

**Figure 1:**
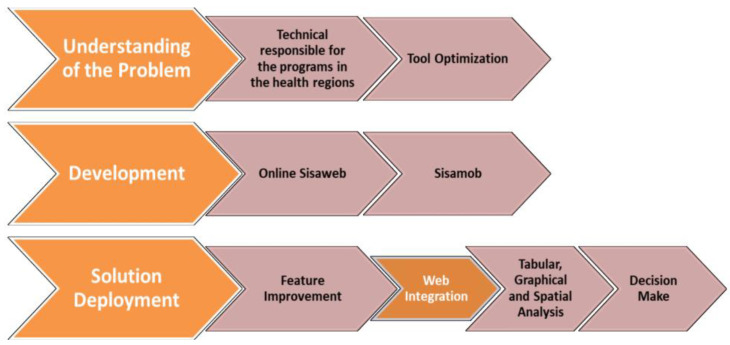
SisaMob application development stages.

The SisaMob application was developed in three stages: 1—Problem Comprehension; 2—Solution Development and Implementation; 3—Solution Impact Evaluation (Figure 1).

### 2.1. Problem Comprehension

For the first stage, it was considered that the activities of urban arboviruses surveillance and vector control were established and consolidated in the SPS, and that the process to compose the strategic information and the way to provide it had already been developed with the implementation of the SisaWeb system by the state and counties.

**Figure 2:**
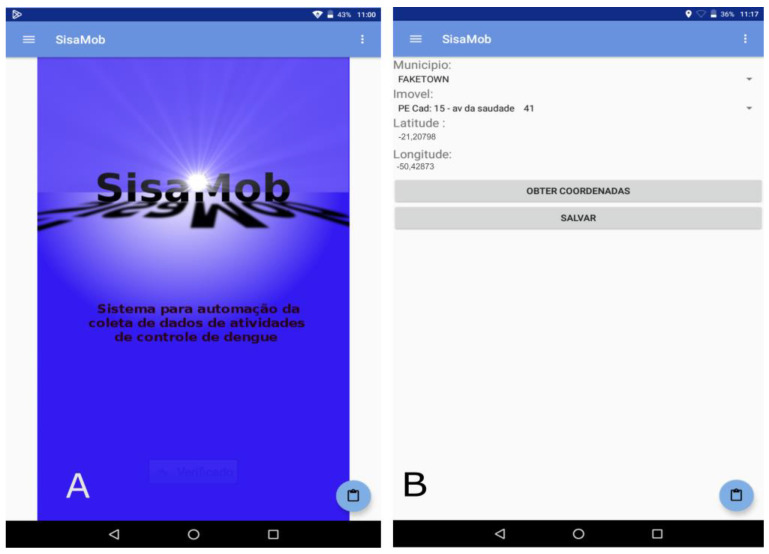
(**A**) Opening screen to application SisaMob; (**B**) Obtaining coordinate by address, with the help of an offline GPS. Note: Sistema de automação da coleta de dados de atividades de controle de dengue = Automated data collection system for dengue control activities; Município = Name of Municipality; Imovel = Property.

The challenge, therefore, was to implement digital data collection. The development of the application (APP), called SisaMob (Figure 2A), took into consideration, at this stage, the field agent work routine. For this reason, the proposed data collection method was the one that caused the minimum impact on the agent’s routine in the field.

This way, the digital collection mirrored the filling out of a paper field form, in which the digital collection of the primary data obeyed the same order as the paper form, following the same instructions of the traditional collecting method, except for the introduction to the initial screen of the collection of geographic coordinates by municipality (“Município”) and property (“Imóvel”) (Figure 2B).

### 2.2. Solution Development and Implementation

With the purpose of providing a solution with a smaller cost for users, the utilization of free software policy was chosen for the development of the application (APP)—SisaMob—in fact not only for the development but also for the use of the systems.

Android was chosen as the operational system. It was conceived inside the development environment “Android Studio”, which is the official development tool adopted by the online services company Google^®^ [6], owner of the “Android” brand. The database was created in PostgreSQL and integrated into the location database in Jason format.

To graphically visualize the geographic database, some polygon-block maps were built, based on the street image from Google Earth^®^ [7], using sectorized information according to the organization of each county in the SPS. In order to build such polygons, with the field map setting the boundaries for each block, the polygon was built with the option to add the polygon from Google Earth^®^ [7], and to each polygon, it was given the corresponding number to the block on the field map. By the end of the task, the polygons were exported to KML format and imported to the Software QGIS^®^ (v. 3.12, 2020) [8], later being exported again to JSON format and incorporated into the SisaWeb system.

**Figure 3:**
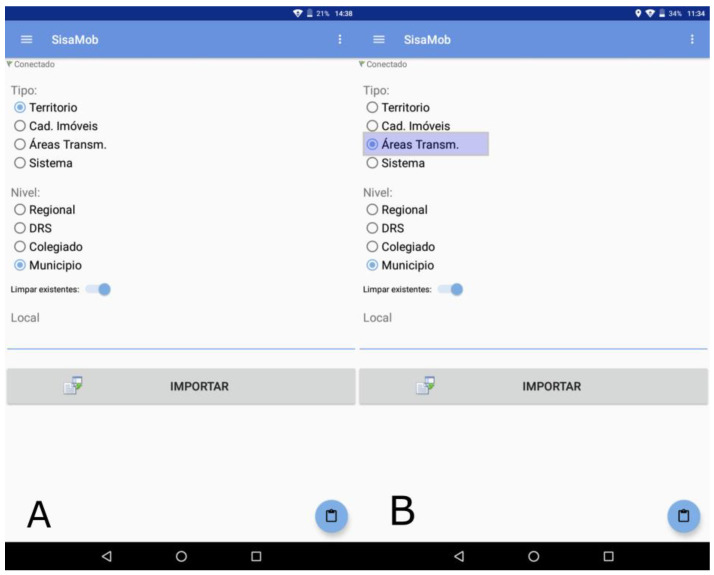
(**A**) APP configuration by hierarchic management level for the implementation of tabular and graphic database of the APP; (**B**) Highlighted the option of transmission areas—it allows the download of the so-called risk areas for implementation of control activities. Note: Tipo = Type; Território = Region; Cad. Imóveis = Property Registration; Áreas Trans. = Transmission Areas; Nível = Level; Regional = Administrative Region; DRS = Administrative Regional Health Board; Colegiado = Collegiate (Set of regional leaders of the state with equal powers); Município = Municipality.

That way, in addition to the geographic coordinates (dots), the data can also be visualized by polygons. One of the advantages of polygon maps is the possibility to define specific areas (priority areas) for specific actions, such as a specific territory to work cases of a disease, for example. Therefore, starting from one case, the system enables the user to select a set of blocks and register it as a “transmission area” (Figure 3B) and, from that area on, visualize specific actions made on these priority areas according to the epidemiologic or entomological situation.

In order to achieve that, there was the purchase of 9-inch “Tablet” devices, provided with an Android operational system, a global positioning system (GPS), and Wi-Fi technology. This configuration enabled incorporating the geographic coordinates of each collection point in the APP in offline mode, dismissing the use of other equipment to collect the coordinates. Likewise, it enabled updating the coordinates when necessary (Figure 2B).

By the end of each day, the data registered in the tablet is synchronized with the SisaWeb through a specific Application Program Interface—API—created for that end and accessed through a Wi-Fi connection. This way, the data are integrated to SisaWeb, where it becomes visible and usable to generate maps, tables, reports, and graphics.

### 2.3. Implementation and Solution Impact Evaluation

The first stage of the process was pre-testing to evaluate the APP, implementing improvements proposed by users, and later implementing the application in all the state of São Paulo. At this stage, regional technicians were capacitated to work with the tool, and they served as multipliers for the field teams. They were trained regarding the equipment functionalities of the SisaMob application, from data entry and checking the information to its eventual transfer to the SisaWeb system.

In the second stage, these multipliers reproduced the training received for the state field teams at the regional level, addressing both theory and practice of using the equipment in the field, checking the information registered by the APP and filling out the paper form. After checking, the information collected by the APP was transferred to the SisaWeb system via Wi-Fi.

In addition to monitoring the use of the APP in the field, a qualitative research was suggested with the objective of evaluating the implantation of the device and the ease of handling the applications by the user, after training with the multipliers. For that, a semi-structured questionnaire was applied, with objective questions scaled from 1 to 5, where 1 was considered bad and 5 was excellent. The quiz was taken using GoogleForms^®^, and the results were compiled into an Excel spreadsheet.

## 3. Results

The functionalities of the SisaMob system were developed in 2018 and implemented in 2019.

**Figure 4:**
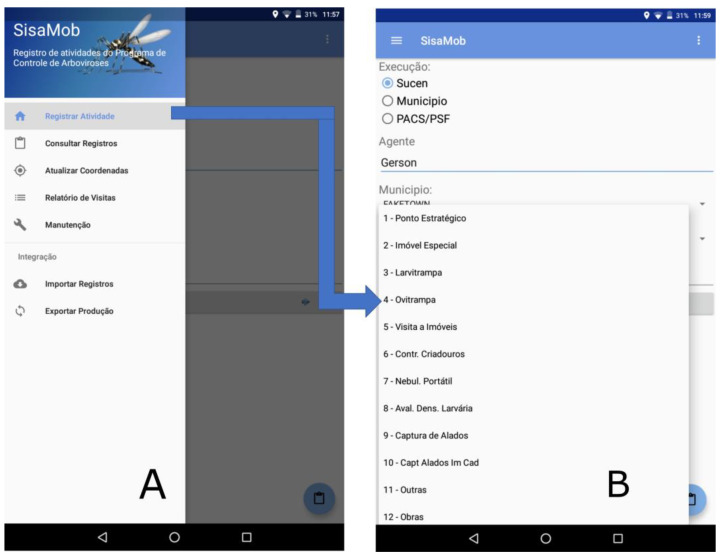
(**A**) Activity selection; (**B**) List of available activities. Evaluation of the implementation of the mobile device and APP. Note: (**A**) Registro de atividades do Programa de Controle de Arboviroses = Record of arbovirus control program activities; Registrar atividade = Record activity; Atualizar coordenada = Update coordinate; Relatório de visitas = Visit Report; Manutenção = Maintenance; Importar Registros = Import Records; Exportar Produção = Export Production. (**B**) Execução: Sucen, Município, PACS/PSF = Execution: Sucen (Superintendence of Control of Endemic Diseases), Municipality, Health Control Agents Program/Agents Family Health Program; Agente = Agent (name of employee); Activities: 1—Estrategic Site; 2—Special Property; 3—Larval Trap; 4—Egg Trap; 5—Visit to Property; 6—Breeding Sites Control; 7—Portable Equipment Nebulization; 8—Larval Density Evaluation; 9—Winged Collection; 10—Winged Collection in Registered Property; 11—Others activities; 12—Collection in Construction Properties.

In order to register the information in the field, it is necessary to download the hierarchical territorial base and the activities from UASVCP (Figure 3A). From then on, the information is filled on staggered processes, identifying the county and activity to be executed, followed by the data collection “house by house”. For each collected point, a set of variables is presented (Figure 4A,B), with the advantage of the automatic collection of geographic coordinates at every new house entry.

Initially, the consistency of the information was evaluated, for both the field reports that were filled, simultaneously with the data entered in the APP through the portable devices and sent to the system. Next, there was the process of information checking.

**Figure 5:**
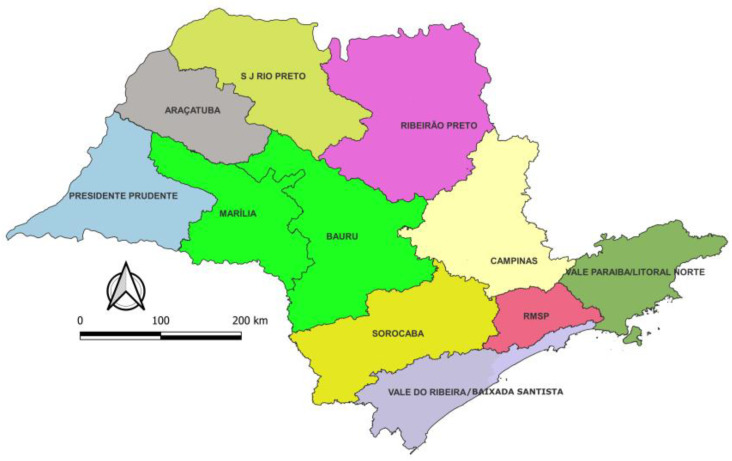
Administrative regions of the state of São Paulo, Brazil.

A focal group was established with the participation of agents from both the state and the counties, who brought suggestions for improvements and other functionality implementations. Among the adjustments needed, not only in the application but also on the web system, some stood out, such as information loss during the transfer process, data duplication, and lack of coordinate information (related to an adjustment to the field equipment). This procedure was followed up for six to eight months, depending on the region of SPS (Figure 5).

After the first adjustments, a new version of the APP was released, and we evaluated the APP functionality through a quiz with 36 employees. They came from different regions of the SPS and from different functions (Table 1: Total I), representing the 10 state regions (Figure 5 and Table 1: Total II).

At all stages of the process, 19.4% of the participants of the survey did not respond to the quiz (Table 2). Among the most common justifications is the fact that it was not their responsibility to perform that part of the process (such as the device configuration, for example), or they did not feel safe, because it was the first time they were making use of that type of equipment or getting in touch with the APP.

Thirty three percent of the people interviewed were responsible for the configuration and installation of the applications (Table 1). According to them, the average time to install the applications was 6 min. Among the ones that described any difficulties at this stage (24 users), 8 (33.3%) reported problems related to the internet, which is a recurrent problem, since there is no policy to provide a free net to the public health system. The institutions make use of their own resources to pay for these kinds of services, which are sometimes of bad quality.

Considering all people interviewed, including the 19.4% (7) that did not respond, we concluded that it was considered adequate (score ≥ 3) for 79.2% (28) of the participants on average (Table 2: Group I). The handling represented 88.9% (32) of the users on average while the ease tablet setup was 69.4% (25) (Table 2: Group I). When asked about the utilization of the APP in the field (Table 2: Group II), 75.0% (27) of the users on average considered it satisfactory or excellent. Some specific points shall be highlighted: regarding “ease of configuring and using of the application SisaMob” and “ease of data entry”, it was considered adequate by 80.6% (29) of users for both questions, and the collection of geographic coordinates represented 77.8% (28) of interviewees. Among the difficulties during this phase of training, the one that appeared the most was the impossibility of capturing the coordinates signal. One of the points negatively evaluated that later served as a parameter for new versions of the applications referred to as “ease to correct the information”, with 38.9% (14) (very unsatisfactory to satisfactory). Regarding replacing the standard form, it was considered satisfactory to excellent by 61.0% (22) of the SisaMob users. Some of the justifications for this rate include: “it was hard to see what was on the device screen” and “it was my first time typing into a tablet”.

It was consensual among the employees that the practical operationalization of the system was a key factor for its acceptance, enhancing the ease to integrate the collected data through the APP with a system that the participants were already familiar with, which also eased its acceptance by operational employees and others.

When presenting the APP to the municipalities, they showed interest due to the ease in handling the APP and the speed in obtaining information. In 2019, about 134/645 (20.8%) of the counties from São Paulo had joined the use of the APP. Nowadays 31.9% (206/645) keep their field teams with the application.

**Figure 6:**
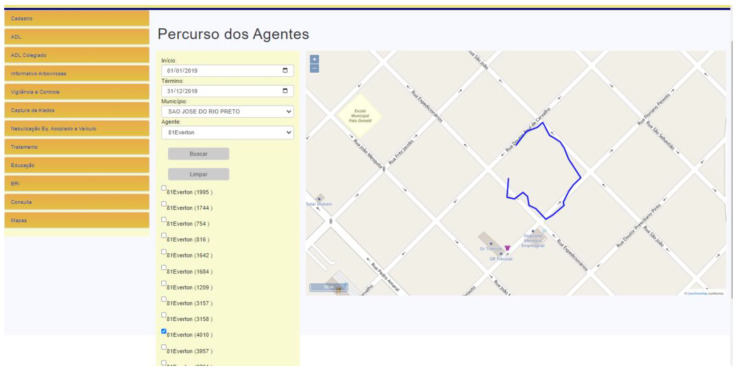
Example of route made by a field agent on a block (blue line). Note: On the left: Menu of activities: Cadastro = Register; ADL = Larval Density Evaluation; ADL Colegiado = Larva Density Evaluation in Collegiate (Set of regional leaders of the state with equal powers); Informativo Arboviroses = Arboviruses Information Report; Vigilância e Controle = Surveillance and Control Activities Report; Captura de Alados = Winged Collection Properties Report; Nebulização Eq. Acoplado a Veiculo = Nebulization with Vehicle-Mounted Equipment Report; Tratamento = Treatment (Report of properties treated with Insecticide—Larvicide or Adulticide); Educação = Educational Activities Report; BRI = Research Results; Consulta = Query; Mapas = Maps. On the Right: Visualization of Agent Route per Period and Agent (Início = Initial Date and Final = Date Final).

The strategy of using tablets speeds up the answer and observation of data, once all is transferred to the server through SisaWeb, and enables the generation of tabular data and graphics. The data can also be visualized by dots and polygons. One of the advantages of polygon maps is the possibility to define specific areas (priority areas) for specific actions, such as a specific territory to work with cases of a disease, for example. Therefore, starting from one case, the system enables the user to select a set of blocks and register it as a “transmission area” (Figure 3B) and, from that area on, visualize specific actions made in these priority areas according to the epidemiologic or entomological situation. SisaWeb offers a menu of options for information management, starting with the user registration, management of information entered or received by SisaMob, surveillance activities and property registration, surveillance activities and searched property control, registration and identification of ovitraps and properties with capture of winged insects, control activities such as vehicular nebulization, educational activities, living conditions evaluations, generation of reports, maps, and even the management of SisaMob (Figure 6: Menu on the left).

The register of geographic coordinates also brings the possibility, inside SisaWeb, to generate a report showing the route of the field agent, based on the activity execution period (Figure 6: on the right). It is possible to follow the activities executed in the field, as well as the operational performance of every agent. The ease to set the coordinate in the tablet with a simple touch allows the field agent to register each visit quickly, which was not possible with the old paper forms.

**Figure 7:**
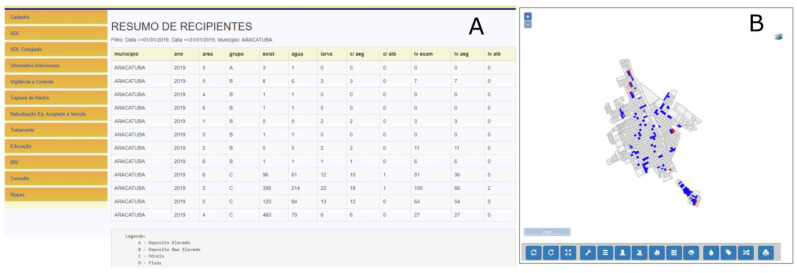
(**A**) Analysis of the local situation in one county: Example summary per group of recipients per area in the city of Araçatuba, SP, Brazil; (**B**) Distribution of blocks with recipients and with recipients containing larva from *Aedes aegypti* (red), Botucatu, SP, Brazil. Note: (**A**) On the left: Menu of activities: Cadastro = Register; ADL = Larval Density Evaluation (Breteau Index—BI); ADL Colegiado = Larva Density Evaluation in Collegiate (Set of regional leaders of the state with equal powers); Informativo Arboviroses = Arboviruses Information Report; Vigilância e Controle = Surveillance and Control Activities Report; Captura de Alados = Winged Collection Properties Report; Nebulização Eq. Acoplado a Veiculo = Nebulization with Vehicle-Mounted Equipment Report; Tratamento = Treatment (Report of properties treated with Insecticide—Larvicide or Adulticide); Educação = Educational Activities Report; BRI = Research Results; Consulta = Query; Mapas = Maps. On the Right: Resumo de recipientes = Breeding sites Summary; Filtro: Data = Filter: Date; Município: Araçatuba= Araçatuba County. Table Header (from left to right): County, Year; Area; Group; Number of existing Containers; Number of Containers with Water, Number of Containers with *Aedes aegypti* Larvae; Number of Containers with *Aedes albopictus* Larvae; Number of Larvae Examined; Number of *Aedes aegypti* Larvae; Number of *Aedes albopictus* Larvae. Legenda = Subtitle: A—High Deposit (Example: water tank); B—Unraised Deposit (Example: cask); C—Mobile containers (Example: can, bottle); D—Fixed Containers (Example: drain).

**Figure 8:**
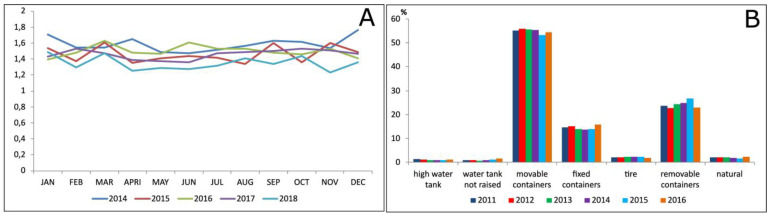
(**A**) Average of recipients with water/property per month by year; (**B**) Percentage distribution of water breeding sites by type and year. State of São Paulo, Brazil.

Another example of the possibilities of systemized information is how detailed it can be. All information is available from the identification of the hierarchic level. On the example showed in Figure 7, the data from a determined period, sorted by group of recipients according to their functions and their positivity on smaller areas inside the county, can be seen. The data are obtained on a tabular (Figure 7A) or graphic form (Figure 7B) and can be handled in other software to generate other analysis that could be considered necessary. The access to the data can be obtained by downloading the files in text format separated by comma (csv), Excel spreadsheets (xls), or even on a PDF (portable document format) report. The example in Figure 8 shows data obtained with the purpose to evaluate the situation in the State of São Paulo regarding the existent recipients. In Figure 8A, the analysis was made by the average of recipients per property per year. On the other hand, Figure 8B brings the frequency of types of recipients per utility per year.

**Figure 9:**
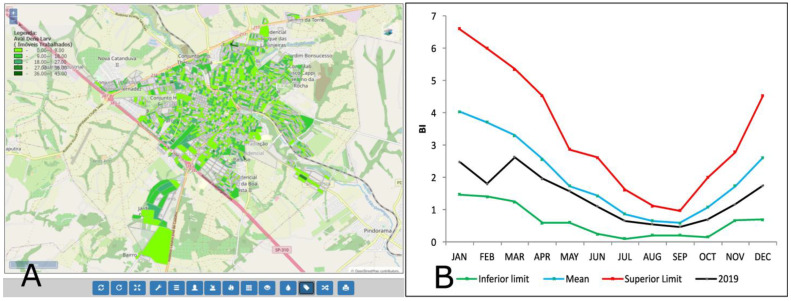
(**A**) Breteau Index—Evaluation of Larval Density in the county of Cantaduva, SP, Brazil, 2019: distribution per block per range of values; (**B**) Control diagram, monthly average rate and standard deviation of Breteau Index (BI) in 2019. Note: Legenda = Subtitle; Aval. Den. Larv (Imóveis Trabalhados) = Larvae Density Index (Collection Properties).

Besides enabling the detailing of the information by hierarchic managing level and by region inside the county, the system allows the information to be presented in different visual formats. Figure 9 shows the data presented spatially (by polygon) and graphically (index values, calculated by the system). This information can be selected from a menu of several variables. In this figure, the evaluation of larval density—Breteau Index in the county of Catanduva, SP, Brazil— the punctual data spatially at the time of measurement and the control diagram are presented. The information enables the manager to evaluate the control activities in their county and where such activities should be implemented or prioritized.

**Figure 10:**
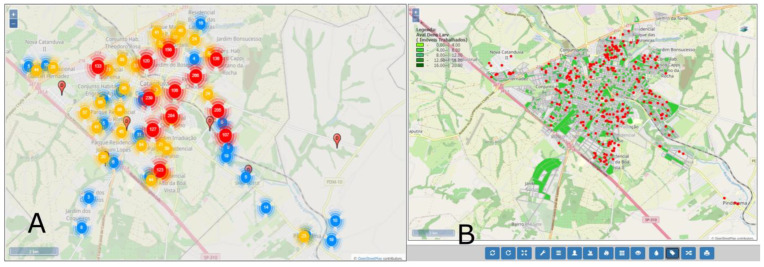
(**A**) Distribution of the number of dengue cases (Source: Sinan) in a district from the county; (**B**) Distribution of dengue cases (red spots) and the range layer from Breteau Index—Evaluation of larval density in the county (green polygons). Note: Legenda = Subtitle; Aval. Den. Larv = Larvae Density Index.

**Figure 11:**
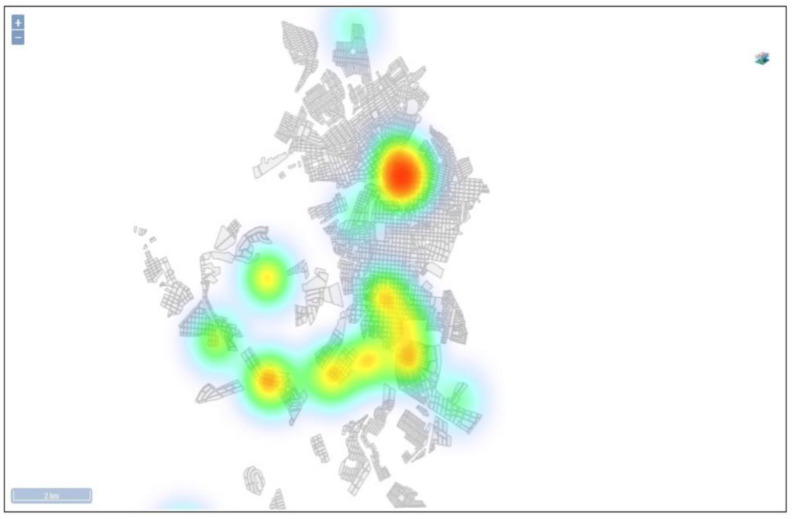
Hot spot map, showing priority areas of dengue transmission.

Another advantage found in SisaWeb is the possibility to import databases from case data [9] and the crossing with the information from the larval density index of *Aedes aegypti* through the creation of layers. Figure 10 shows the number of cases of dengue in the county (Figure 10A) and the number of cases of dengue with the value range layer from the Breteau Index in the county (Figure 10B). As mentioned before, it is possible to rapidly obtain indexes by the ease of the collected information being available only with synchronization from the APP with the system, besides the possibility to cross the information with other databases. It is also possible to make simpler analyses, such as hot spots, for the definition of priority areas (Figure 11).

## 4. Discussion

Since the 1970s, a growing integration between technology and the health area can be observed, and, nowadays, the use and development of software is a part of many people’s daily life in health departments. Any process becomes almost impracticable without the use of computerized systems, since they increasingly support in the search for strategies to plan and structure actions to find solutions through the collected data. That happens not only at the management level but also at the clinical level [10]. According to Kraus-Silva (2004) [11], the health technological assessment in developed countries presents itself more and more as a subsidy to process planning and evaluate services and programs.

Currently, the importance of information is a fact widely discussed and accepted and should compose the available arsenal for decision-making and the definition of strategies for disease control. Digital transformation already became a reality as nowadays many human activities are being processed in this technological universe. Likewise, the technology for mobile devices is an activity of great interest and is rapidly growing among researchers [12]. Whilst private sector companies emerge with large investments in information technology, the public sector is still in its first steps in this direction. There is still a lot to be invested, especially in the public health area.

Concerning the public service, the information is the instrument that takes managers to plan investments, especially to provide human and financial resources. However, in the public health area, mainly when it comes to the control of vector-borne diseases, the faster the information is made available, the better is the response with an action that will be implemented in the risk area or during the transmission in course.

The mobile applications for digital collection of health data, especially in the research field, have gained substantial attention in the last decade, becoming an important tool to increase the collection efficiency [12,13,14,15,16,17,18]. These systems tend to be relatively simple to develop and maintain, besides minimizing the time between the initial collection and the communication of the data to researchers and interested parts [18].

The challenge in collecting the data is to keep them precise, using strategies as a unified collecting and storage system as well as restricted value entry fields in the collecting data forms, which helps to maintain this precision. Another point is the need to perform routine data integrity checks throughout collection data activities in order to minimize errors. According to Elson et al. (2022) [18], among the lessons learned with the use of data collection through mobile devices is that, when implementing these strategies, it is important to balance the flexibility for users with measures to control the quality, in order to reduce mistakes.

Before the development of digital collection, the process of data collection in the UASVCP and other surveillance and control programs in the state of São Paulo was made on paper forms. The bulletin was filled in by the field agent, and a supervisor had to check the information; only then it was forwarded to be entered to the web system. Depending on the quantity of primary data generated in the field, it could take at least a week to have these data completely entered into the system, to be checked and consolidated later, and only then to generate the information.

The use of mobile devices enabled the reduction in the data filling time. The use of restricted and objective fields allowed the user to select the variable with only a touch, and then it was only necessary to type a number to quantify the variable. It is important to mention, though, that the adoption of geographic coordinates collection by the APP gave agility to the system, excluding the stage of writing down addresses in the field, typing and later geo-codifying the addresses, and minimizing the errors annotated on the field, which meant a great innovation.

Other advantages observed with the use of SisaMob in a portable device are the following: 1—the user does not need to have computer knowledge to fill in the form or to handle the database to make the evaluations; 2—registering the data can be performed offline, reducing the cost since it does not need a mobile data network; 3—only when the data collection is finished, the information saved in the equipment is uploaded through Wi-Fi on the same day it was collected, generating the data in a timely manner, as said before, and eliminating the stage of typing with the reduction in the occurrence of annotation or typing errors; 4—the automation in obtaining geographic coordinates enables the system to calculate the duration of each visit, which was not possible using the paper forms; 5—as the system integrates the database, it was possible to reduce the time to obtain the results of the culicids identification. In this case, a technician trained to use the system can access the system and enter the laboratory results of the analyzed sample, needing for that just a computer with access to the internet; 6—the other advantage was the construction and free availability of the geographic base of the blocks, made by our Institutional Group, allowing this database to be available for other systems being developed by the group. SisaWeb was developed according to the content that was already used by the state and the counties, and SisaMob was developed by the same logic used in the paper forms. The pre-testing and the discussion with the technicians at state level, which already worked with UASVCP, made the development and acceptance of the APP much easier. The systems feedback from the faults, identified by the users, enabled a rapid improvement in the performance, along with the advantage of having in the team an information technology specialist.

The implementation of the digital data collection covered 100% of the vector control state teams responsible for executing entomological assessment activities in 20.8% of the counties in 2019, when the APP became available. However, 31.9% of counties that joined the device were still incipient in 2022. Nevertheless, medium and large cities (with over 50 thousand people) are part of this list, and these are the ones that create the biggest data amount. The counties face some difficulties, such as adequate maintenance of the equipment by the field professional, and the high rate of replacement of these professionals, which brings the need for periodic training. It is also important to mention that the two years of restrictions due to the COVID-19 pandemic resulted in the destructuring of the teams in many counties.

In that regard, managers have been encouraged to join and adopt the use of the equipment since the sate provides the applications integrated to the database for free. In the case of UASVCP, all the information collected is made by the counties and will be fed back by them. This way, the state offers the APP without the need to invest in its development and only guarantees the uniformity of the utilized solutions, training, and technical support.

It is to be emphasized, as previously observed, that this is a continuous process and will have constant feedback due to the premise that access to reliable epidemiologic and entomologic information at the right time can support the decision-making process on different hierarchic levels. This need is being felt in many countries that have been developing geographic information systems but often make use of more expensive and difficult handling instruments. This requires qualified personnel with great knowledge in specific areas, and sometimes they use commercial software to make their data analysis, which makes the operation even more expensive, since it requires license acquisitions which make it impossible to merge the information so that it can be compared on different hierarchic levels [18,19,20,21,22].

This APP is the first application for routine surveillance and vector control integrated with the vector-borne disease database aimed at state municipal managers in Brazil. Other applications successfully used were for managing outbreaks or restricted to research, such as SORMAS [23], for data collection, situation assessment, and coordination of response measures in outbreak surveillance processes, as well as the mHAT, concerning a study for the surveillance of malaria cases in remote communities [24].

Duncombe et al. (2012) [25], in their review about the use of geographic information systems on dengue surveillance, discuss the need to identify variables that can explain the transmission of dengue fever and emphasize that the use of a geographic information system, especially in initial stages, could be a useful tool to visualize the relation amongst the variables and to establish cluster zones where the public managers should take priority actions. With that in mind, the integration between SisaMob and SisaWeb provides tools to the public sector to quickly visualize epidemiological, entomological and operational data.

The advance in the construction of the SisaWeb information system—SisaMob—allowed the universalization of the information, since the data are available on report mode for public consultation through dynamic reports in which the user selects the variables with ease. That integration represents an enhancement in the quality of the collected data and allows a better direction of control at the local level.

Much can still be implemented in SisaWeb, such as the integration with other health bases through a BigData that could possibly enlarge the analysis with the data from the demographic profile of the population at risk, environmental conditions, useless collection points such as Eco Points [26], or even climate variables, both for monitoring the spread of *Aedes aegypti* and the dengue virus and other urban arboviruses. Nevertheless, with this system, it was already possible to obtain a detailed overview of the entomologic and epidemiologic situation, making it possible to analyze the indexes behavior at different levels of aggregation, supplying tabular data and graphics in order to monitor these indexes.

Currently, data collection systems through portable devices are recognized as important tools and have been more and more utilized in events related to health, even if it is to collect clinical data [12,13,18] or to vector control [19,20,21]. They are recognized by the World Health Assembly, which approved by unanimity a resolution recognizing its potential to help achieve the United Nations Sustainable Development Goals, which specifically include vector-borne diseases [27].

There are still challenges to be overcome, such as mechanisms for evaluating the efficacy and effectiveness of the information generated in the field, improving the quality of the information, increasing the potentiality of the tool to produce robust analysis, and constructing different indexes that could direct the actions in a more efficient way. However, it must be considered that investments in human resources are needed to make projects and implement and manage the systems of high-quality data collection.

It is still worth mentioning that there is the possibility of providing the system to different regions outside the state, if the UASVCP guidelines are kept the same and there is a host for the system and a technician for the management. Lastly, this same logic is being used by our team and provided to other programs of Surveillance and Vector Control, such as Chagas disease, Schistosomiasis mansoni, and for the collection of Culicidae and wild sandflies, as well as for the Harmful and Venomous Arthropods Surveillance and Control Program.

## Figures and Tables

**Table 1:** Characterization of the participants for the evaluation of the implementation of SisaMob in the Superintendence of Endemic Disease Control (current Pasteur Institute), 2019.

Interviewee Characteristics	N	%
**I—Activity Function**		
Field Agent (N = 100) *	22	61.1
Head of Technical Section and Administrative Officer (N = 15) *	12	33.3
Health Technical Director (N = 10) *	2	5.6
**Total I**	**36**	**100.0**
**II—Region of Origin**		
1—Metropolitan Region of São Paulo	4	11.1
2—Baixada Santista / Ribeira Valley	2	5.6
3—Paraíba Valley/North Coast	3	8.3
4—Sorocaba	3	8.3
5—Campinas	5	13.9
6—Ribeirão Preto	3	8.3
7—São José do Rio Preto	3	8.3
8—Araçatuba	7	19.4
9—Presidente Prudente	3	8.3
10—Marília/ Bauru	3	8.3
**Total II**	**36**	**100.0**

* Number of employees according to activity function.

**Table 2:** Number of participants evaluated and average and percentual for each subgroup, according to questioned criteria and evaluation rate.

Question	1	2	3	4	5	Did Not Answer	Total
SCORE							
**I—Mobile Device Handling**							
Ease of tablet setup		0	9	6	10	11	36
Ease of use of the tablet		1	9	12	11	3	36
**Average Group I**		**0.5**	**9.0**	**9.0**	**10.5**	**7.0**	**36.0**
**%**		**1.4**	**25.0**	**25.0**	**29.2**	**19.4**	**100.0**
**II—SisaMob**							
Ease of configuring and using the APP			7	13	9	7	36
Ease of data entry	1		9	13	7	7	36
Ease of correction of the information collected in the field	1	1	12	9	6	7	36
Compared with the bulletin, replaced standard form		6	5	6	11	7	36
Degree of ease in collecting coordinates		1	8	10	10	7	36
**Average Group II**	**0.4**	**1.6**	**8.2**	**10.2**	**8.6**	**7.0**	**36.0**
**%**	**11.6**	**4.4**	**22.8**	**28.3**	**23.8**	**19.4**	**100.0**

## Data Availability

The reports and maps of the SisaWeb system can be accessed at the links https://vigent.saude.sp.gov.br/sisamap (accessed on 10 April 2023) and https://vigent.saude.sp.gov.br/sisawebinfo (accessed on 10 April 2023). Individual data are not shared publicly and can be made available for research, as long as they request access based on the General Data Protection Law. See details at https://www.gov.br/cidadania/pt-br/acesso-a-informacao/lgpd (accessed on 10 April 2023).
